# *echinus*, required for interommatidial cell sorting and cell death in the *Drosophila *pupal retina, encodes a protein with homology to ubiquitin-specific proteases

**DOI:** 10.1186/1471-213X-7-82

**Published:** 2007-07-05

**Authors:** Jeffrey M Copeland, Ian Bosdet, J Douglas Freeman, Ming Guo, Sharon M Gorski, Bruce A Hay

**Affiliations:** 1 Division of Biology, MC 156-29, California Institute of Technology, Pasadena, CA 91125, USA; 2 Genome Sciences Centre, British Columbia Cancer Agency. Vancouver, British Columbia V5Z 1L3, Canada; 3 Department of Neurology, Brain Research Institute, The David Geffen School of Medicine at UCLA, Los Angeles, CA 90095, USA

## Abstract

**Background:**

Programmed cell death is used to remove excess cells between ommatidia in the *Drosophila *pupal retina. This death is required to establish the crystalline, hexagonal packing of ommatidia that characterizes the adult fly eye. In previously described *echinus *mutants, interommatidial cell sorting, which precedes cell death, occurred relatively normally. Interommatidial cell death was partially suppressed, resulting in adult eyes that contained excess pigment cells, and in which ommatidia were mildly disordered. These results have suggested that *echinus *functions in the pupal retina primarily to promote interommatidial cell death.

**Results:**

We generated a number of new *echinu*s alleles, some likely null mutants. Analysis of these alleles provides evidence that *echinus *has roles in cell sorting as well as cell death. *echinus *encodes a protein with homology to ubiquitin-specific proteases. These proteins cleave ubiquitin-conjugated proteins at the ubiquitin C-terminus. The *echinus *locus encodes multiple splice forms, including two proteins that lack residues thought to be critical for deubiquitination activity. Surprisingly, ubiquitous expression in the eye of versions of Echinus that lack residues critical for ubiquitin specific protease activity, as well as a version predicted to be functional, rescue the *echinus *loss-of-function phenotype. Finally, genetic interactions were not detected between *echinus *loss and gain-of-function and a number of known apoptotic regulators. These include Notch, EGFR, the caspases Dronc, Drice, Dcp-1, Dream, the caspase activators, Rpr, Hid, and Grim, the caspase inhibitor DIAP1, and Lozenge or Klumpfuss.

**Conclusion:**

The *echinus *locus encodes multiple splice forms of a protein with homology to ubiquitin-specific proteases, but protease activity is unlikely to be required for *echinus *function, at least when *echinus *is overexpressed. Characterization of likely *echinus *null alleles and genetic interactions suggests that *echinus *acts at a novel point(s) to regulate interommatidial cell sorting and/or cell death in the fly eye.

## Background

The adult *Drosophila *eye consists of 750–800 individual unit eyes, known as ommatidia, which are arranged in a hexagonal lattice. Each ommatidium consists of 8 photoreceptors, 4 lens-secreting cone cells and 2 primary pigment cells. Ommatidia are separated from each other by secondary and tertiary (2° and 3°) pigment cells, and by sensory bristles. Each of these cell types occupies a stereotypic position within the lattice. Pattern formation in the eye is initiated in the 3rd larval instar as a wave of morphogenesis sweeps across the epithelial cell layer in the eye imaginal disc. First, eight photoreceptor cells and four lens-secreting cone cells are specified through sequential inductive interactions. During early pupal stages, cone cells come to cover the photoreceptors. They also recruit two primary pigment cells, which surround the cone cells. Cells that have not been specified at this stage form the interommatidial cell (IOC) lattice, which will ultimately be composed of secondary pigment cells, tertiary pigment cells, and bristles. These cells initially appear undifferentiated and unpatterned, with several layers of IOCs often separating neighboring ommatidia. Reorganization begins with presumptive lattice cells maximizing their contacts with primary pigment cells rather than with other lattice cells. This results in each lattice cell being connecting to at least two primary pigment cells, and with each ommatidia being separated by a single layer of lattice cells, arranged in an end-to-end chain. About two-thirds of these cells will go on to develop as secondary pigment cells, each of which makes up one face of the ommatidial hexagon, or tertiary pigment cells, which make up alternative vertices, with bristle groups making up the other vertices. The remainder of the IOCs are eliminated by apoptotic cell death [1,2].

Much cell death in *Drosophila *takes the form of apoptosis [3]. Caspase proteases are the central executioners of apoptotic cell death [4]. Dronc is required for many cell deaths in the fly [5-8], including those of the IOCs [9]. Once activated through interactions with the adaptor Ark, Dronc cleaves and activates effector caspases such as Drice and Dcp-1 that are thought to bring about cell death [5,6]. Drice is activated during the stages in which IOC death occurs [10], and Drice mutants lack some, but not all, IOC death, highlighting the importance of this protease [11,12]. DIAP1 is a cell death inhibitor that suppresses the activity of Dronc and caspases activated by Dronc through several different mechanisms [5,6,13-19]. Reaper (Rpr) [20], Head involution defective (Hid) [21], Grim [22], Sickle [23-25], and Jafrac2 [26], known collectively as the RHG proteins after their founding members Rpr, Hid and Grim, bind to DIAP1 through a short-N-terminal motif and disrupt DIAP1-caspase interactions through several mechanisms, each of which has the effect of unleashing a cascade of apoptosis-inducing caspase activity. Flies that lack Hid show defects in Drice activation and IOC cell death [10,27], while mutants for the other proteins are not available. Together these observations suggest that IOC death is driven, at least in part, by Hid-dependent inhibition of DIAP1, which facilitates activation of Dronc and Drice (Fig. 4 schematic).

Ubiquitination, and thus presumably deubiquitination, plays several important roles in the regulation of this cell death pathway. DIAP1 is an E3 ubiquitin ligase [28-32] that can ubiquitinate and inactivate Dronc [15,16]. DIAP1 can also promote the ubiquitination and degradation of other pro-apoptotic proteins that it binds such as Reaper [33]. DIAP1 also ubiquitinates itself [28-32,34] and DIAP1 ubiquitination can be stimulated by the RHG proteins [28-32,34]. Many components of the ubiquitin pathway have been identified as regulators of RHG-mediated cell death. Examples include the ubiquitin activating enzyme (*uba1*), two components of an SCF-type E3 ubiquitin ligase (*skpA *and a novel F-box gene, *morgue*) and the deubiquitinating enzyme *fat facets *[29,31,32]. However, the points at which these proteins work to regulate death are largely unknown.

As noted above, the IOCs are initially arranged in double or triple rows between ommatidia in the pupal eye. IOCs then rearrange in an end-to end configuration to form a single layer or row of cells separating primary pigment cells of different ommatidia. Cell death only occurs after this rearrangement, or sorting, is complete. A key player in this process is the immunoglobulin family protein Roughest (Rst). In the absence of roughest the IOCs remain stacked side-by-side in multiple rows and IOC death does not occur [2]. Rst is localized in IOCs to the border between IOCs and primaries [35], in a process that requires DE-cadherin and Notch [36-38]. Recent observations suggest that Rst promotes sorting by physically interacting with Hibris, another immunoglobulin family membrane protein expressed in primary pigment cells that is also required for IOC sorting and death [37].

The EGF receptor pathway provides important anti-apoptotic signals to IOCs. Loss of EGFR signaling in the pupal eye results in fewer IOCs [39], while activation of EGFR or Ras promotes IOC survival [40]. EGFR activation promotes IOC survival at least in part by negatively regulating *hid *levels and pro-apoptotic activity [10,27,41]. Pro-survival signaling through the EGFR is antagonized by Notch-mediated signals (probably between IOCs), which are required to remove excess IOCs [10,40]. The amount of contact an IOC has with primary pigment cells (which produce EGFR-dependent survival signals) as opposed to other IOCs (which produce Notch-mediated signals that antagonise the EGFR pathway) is likely to be an important part of the calculus that determines IOC fate. Ubiquitination plays important roles in regulating both signaling pathways. The EGFR is monoubiquitinated following ligand binding, and this promotes receptor endocytosis and degradation, thus attenuating signaling [42]. In the Notch pathway, monoubiquitination of both ligands and Notch by multiple E3 ligases is associated with endocytic events that promote signaling. Ubiquitination of Notch by other E3 ligases is associated with internalization and lysosomal degradation [43].

Other proteins that regulate IOC survival have been identified. The Runx DNA-binding protein Lozenge is required for IOC death [44-46]. Lozenge pro-apoptotic activity is mediated by its ability to induce the expression of Argos, a secreted inhibitor of EGFR activation in cone cells, and 2° and 3° cells [44]. Lozenge also activates expression of Klumpfuss, a transcription factor with similarity to the Wilm's Tumor suppressor, in 2° and 3° cells [44]. Klumpfuss function is required for normal levels of IOC death, and genetic evidence suggests that it antagonizes EGFR signaling downstream of receptor activation [47].

*echinus *(*ec*), defined by a single allele, *ec*^1^, was identified by Calvin Bridges in 1918 as a X-chromosome-linked, recessive, rough eye mutant (as described in [48]). More recently, Wolff and Ready showed that *ec*^1 ^flies had decreased levels of IOC death, much like *rst *mutants. However, while IOC sorting failed to occur in *rst *animals, sorting was largely (though not completely) intact in *ec*^1 ^flies [2,49]. Expression of the baculovirus caspase inhibitor p35 also prevents death but not sorting, giving rise to a pupal retinal phenotype with many similarities to (though not identical to) that observed in *ec*^1 ^flies [50]. Together these observations have suggested that *ec *acts primarily subsequent to sorting, perhaps regulating cell death directly [49-51]. To understand the role *echinus *plays in bringing about IOC death we generated a number of new *echinus *alleles and re-examined phenotypes associated with *ec*^1 ^following extensive outcrossing to remove modifiers. Multiple alleles, including putative null alleles, show defects in cell sorting as well as cell death. We cloned the *echinus *locus and found that it encodes multiple isoforms of a protein with homology to the ubiquitin specific protease (USP) family of proteases. Somewhat unexpectedly, versions of Echinus that lack residues thought to be important for USP catalytic activity can rescue the *echinus *loss-of-function sorting and cell death phenotypes. We were unable to detect significant interactions between loss- and gain-of-function *echinus *alleles and a number of known or suspected regulators of IOC death. *echinus *may primarily regulate cell sorting, with loss of death being only a consequence of an earlier defect in this process. Alternatively, *echinus *may regulate cell sorting and cell death, with regulation of death occurring at a novel point, perhaps through mechanisms that are independent of USP activity.

## Results

### CG2904 encodes echinus, which is expressed at low levels ubiquitously in the pupal retina

**Figure 1:**
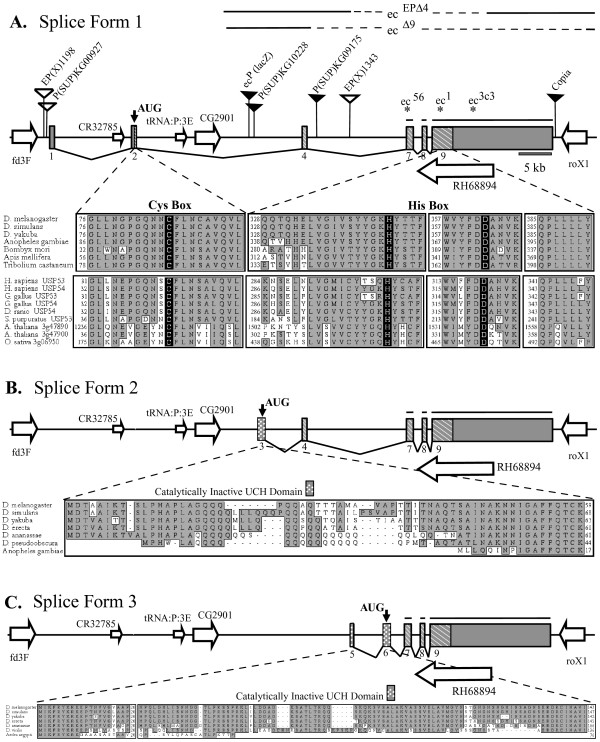
*echinus *gene structure, mutants and surrounding genomic region. Echinus (CG2904) exons are indicated by shaded boxes. Exons are numbered sequentially with respect to the 5' end of the gene. The shaded boxes with diagonal lines represent the conserved USP domain. Three different splice versions identified through cDNA sequencing are illustrated in panels A-C. Exons common to all three splice versions are noted with lines above numbered exons 7,8, and 9. Nearby genes are indicated (open arrows), as are Flybase annotated P element insertions, and *ec*^*PlacZ*^. Open triangles indicate P element insertions that are wildtype with respect to *echinus*, while filled triangles indicate P element insertion lines with rough eyes that fail to complement *echinus*. The location of EMS-induced point mutations in *echinus*, *ec*^56 ^and *ec*^3*c*3^, and mutations identified in *ec*^1 ^(a point mutation and a Copia insertion) are indicated by asterisks. The locations of the breakpoints for the *echinus *deletion alleles *ec*^*EP*Δ4 ^and *ec*^Δ9 ^are indicated by dotted lines at the top. (A) ec-SF1 encodes a version of *echinus *that contains Cys and His box residues important for USP catalysis, as noted by the highlighted residues. These and surrounding sequences are highly conserved in predicted *echinus *homologs in other insect species. Highly related sequences are also found in a number of other species. (B) The ec-SF2 transcript initiates at a downstream exon, which contains an initial methionine and coding sequences that lack a Cys box. The 3' exon containing His box sequences (exon 9) is still present. (C) The ec-SF3 transcript initiates at a distinct position further 3', and also contains His box sequences, but lacks Cys box sequences. Sequences highly related to this alternative N-terminus are also found in a number of other species.

As a first step to cloning the *echinus *locus we used both EMS mutagenesis and P element excision to generate new *echinus *alleles. We identified an X chromosome-linked P element insertion line, *ec*^*PlacZ*^, with a recessive rough eye phenotype that failed to complement *ec*^1 ^(Fig. 1A). We generated a number of excisions of this element. The rough eye phenotype was reverted in some of these, indicating that the P element insertion was responsible for the *echinus *phenotype. The *ec*^*PlacZ *^transposon is located between CG2901 and CG2904, suggesting one or the other of these genes as good candidates to encode *echinus*.

**Figure 2:**
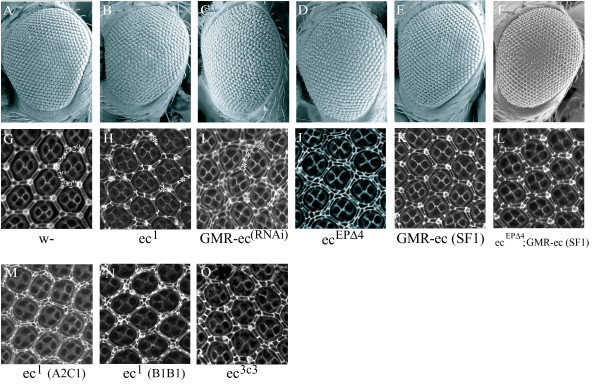
Flies with mutations in CG2904 have rough eyes, defects in IOC sorting, an increase in IOC number (A-F) SEM views of adult fly eyes of various genotypes. (G-O) Pupal retinas of various genotypes stained with anti-Dlg. (A, G) Wildtype flies have regularly spaced ommatidia and an invariant number of IOCs. Cell types indicated are bristle (B), 2°, 3°, and asterisk represent extra IOCs. (B,H) *ec*^1 ^flies obtained from the Bloomington Stock center have rough eyes and a modest number of extra 2° and 3° pigment cells. (C,I) GMR-driven RNAi of CG2904 results in flies with rough eyes and a large increase in IOCs, with many stacked side-by-side in parallel rows. (D,J) Flies homozygous for a deletion in CG2904, *ec*^*EP*Δ4^, have rough eyes, a large increase in IOCs, with many cells stacked side-by-side in parallel rows. (E,K) GMR-dependent expression of ec-SF1 has no effect on the adult eye and does not cause any excess death of IOCs. (F,L) Expression of GMR-ec-SF1 restores normal levels of IOC death to *ec*^*EP*Δ4 ^flies. (M,N) Pupal eyes from two independent stocks of *ec*^1 ^outcrossed for 5 generations. There are increased numbers of IOCs as compared with the original *ec*^1 ^stock, and many extra cells are aligned side-by-side in parallel rows. (O) Pupal eyes from *ec*^3*c*3 ^flies have a modest increase in IOC number and few defects in cell sorting.

Ommatidia from wildtype flies are arranged in a regular hexagonal pattern (Fig. 2A), and extra IOCs are not observed in pupal eyes (Fig. 2G; Table 1). In contrast, adult eyes of *ec*^1 ^flies are rough (Fig. 2B), and extra IOCs are present in the pupal retinas (Fig. 2H). To determine if CG2904 encodes *echinus *we generated flies expressing dsRNA corresponding to CG2904 in order to trigger RNAi-dependent knockdown of CG2904 expression, under the control of the eye-specific-GMR promoter (GMR-CG2904-RNAi). Consistent with this hypothesis, GMR-CG2904-RNAi flies had a rough eye phenotype (Fig. 2C) and extra IOCs (Fig. 2I). To create deletion alleles of CG2904 we generated excisions from a nearby P element insertion line, *EP(X)1343*, that is wildtype with respect to *echinus *(Fig. 1A). Multiple excision lines were identified that had rough eyes as homozygotes. Each of these failed to complement *ec*^1 ^or *ec*^*PlacZ*^. Breakpoints for four of these were determined, and each was found to delete sequences within the CG2904 transcript. We have focused our analysis on one of these, designated *ec*^*EP*Δ4 ^(Fig. 1A; Fig 1D, J). Pupal eyes from *ec*^*EP*Δ4 ^also showed extra IOCs (Fig. 2J). We found that the CG2904 gene was also disrupted by the breakpoints of an excision allele generated from an alternate P element insertion (*ec*^Δ9^; Figure 1H. Kramer, unpublished). This mutant also had rough eyes and extra IOCs (data not shown). More recently, a number of new P element insertion lines in the surrounding genomic region have been identified [52]. Several have a rough eye phenotype that fails to complement *ec*^1^, and each of these is located near CG2904 (Figure 1A). We also identified several new EMS alleles of *echinus *(see methods for details). We sequenced CG2904 coding sequences from one of these and identified an E125-Stop change in *ec*^56 ^(Figure 1A). We also identified a stop mutation (L792-Stop in splice form 1) in the EMS-derived *ec*^3*c*3 ^allele [53]. Finally, we sequenced CG2904 coding and nearby regions from the original *ec*^1 ^stock. Two significant alterations were noted: an R295-Stop mutation in the coding region, and a Copia element inserted 3' to the transcript. TUNEL assays and/or anti-active caspase immunostaining conducted using *ec*^Δ9^, *ec*^*EP*Δ4^, *ec*^*PlacZ*^, and *ec*^56^, confirmed a reduction in apoptosis in mid-pupal retinas (see Additional File 1).

**Table 1:** Average Number of IOCs

Genotype	Average no. IOC
w-	9.0
ec^1^	10.6 ± 1.5
GMR-ec^RNAi^	14.1 ± 2.1
ec^EPΔ4^	14.1 ± 1.7
GMR-ec (SF1)	9.1 ± 0.3
ec^EPΔ4^; GMR-ec (SF1)	9.6 ± 1.0
ec^1 ^(A2C1)	14.8 ± 1.8
ec^1 ^(B1B1)	14.4 ± 2.2
ec^3c3^	10.6 ± 1.0

Table 1 shows the average number of IOCs for the genotypes indicated. Examples of adult and pupal eyes from these genotypes are shown in Fig. 2.

Together, the above results strongly suggest that CG2904 is *echinus*. To test this definitively we asked if expression of CG2904 could rescue the *echinus *phenotype. We identified several cDNAs for CG2904 from a larval-pupal library (see below). One of these, designated ec-SF1, was introduced into flies under the control of the GMR promoter (GMR-ec-SF1 flies). These flies have wildtype-appearing eyes and IOC number (Fig. 2E,K), but when introduced into the *echinus ec*^*EP*Δ4 ^background, GMR-ec-SF1 restored adult ommatidial patterning and normal IOC cell death (Figure 2F,L). RH68894 represents a group of 3 kb cDNA species that overlap echinus on the anti-sense DNA strand. RH68894 is predicted to be a non-coding RNA, based on the lack of any reading frame of significant size. To test whether RH68894 has any role in mitigating the echinus phenotype, GMR-RH68894 was introduced into the fly (GMR-RH68894 flies). Adult eyes of GMR-RH68894 flies appear wildtype. In addition, when introduced into *ec*^*EP*Δ4^, GMR-RH68894 failed to rescue or alter the echinus rough eye phenotype. Together these observations demonstrate that CG2904 (hereafter simply referred to as *echinus*), and not RH68894, encodes *echinus*.

Pupal retinas from animals homozygous for new alleles of *echinus *such as *ec*^*EP*Δ4^, *ec*^Δ9 ^and *ec*^56^, as well as those from wildtype flies expressing GMR-CG2904-RNAi, showed a striking difference from retinas mutant for *ec*^1 ^(as obtained from the Bloomington Stock Center). *ec*^1 ^pupal eyes showed a significant increase in IOC number subsequent to the time when death normally occurs, and this increase was associated with only a modest level of side-by-side alignment of IOCs (Fig. 2; Table 1) [2]. One of the new EMS alleles isolated, *ec*^3*c*3^, which contains a stop codon near the C-terminus of the *echinus *coding region, showed a similar phenotype (Fig. 2O). ec^3c3 ^is likely to be a partial loss-of-function allele of *echinus *since *ec*^3*c*3^/Df(1)HC244 results in a stronger adult rough eye phenotype than that observed in homozygous *ec*^3*c*3 ^flies (see Additional File 2). In contrast, pupal eyes from *ec*^*EP*Δ4 ^and GMR-CG2904-RNAi (Fig. 2), *ec*^Δ9 ^and *ec*^56 ^(data not shown) showed a greatly increased number of IOCs (Table 1) and many of these extra cells were aligned side-by-side. The Bloomington *ec*^1 ^stock was outcrossed to wildtype flies for 5 generations in two independent experiments. Interestingly, pupal eyes from both of these outcrossed lines showed an increase in the number of extra IOCs (Table 1) and IOC cell stacking (Fig. 2M,N). Importantly, both sorting and death phenotypes were rescued by GMR-dependent expression of ec-SF1 for multiple *echinus *alleles (Fig. 2). Together, these observations suggest that the original *ec*^1 ^line has picked up one or more suppressor mutations and that the true *echinus *null phenotype in the pupal eye results in extra IOCs, with many of these cells being stacked side-by-side. In addition, there seems to be a direct correlation between the severity of defects in sorting and those in cell death. Thus, pupal retinas from the original *ec*^1 ^allele and *ec*^3*c*3 ^displayed mild defects in sorting and IOC death, while retinas from the deletion allele *ec*^*EP*Δ4^, outcrossed *ec*^1^, GMR-CG2904-RNAi, and *ec*^*EP*Δ4^/Df(1)HC244 (a deficiency which covers the *echinus *locus) (data not shown), displayed much more severe defects in sorting and IOC death. These observations raise a question as to whether the *echinus *decrease-in-IOC death phenotype is a result of loss of *echinus *function as a death activator, or a secondary consequence of a failure in sorting, which precludes death signaling (discussed further below).

**Figure 3:**
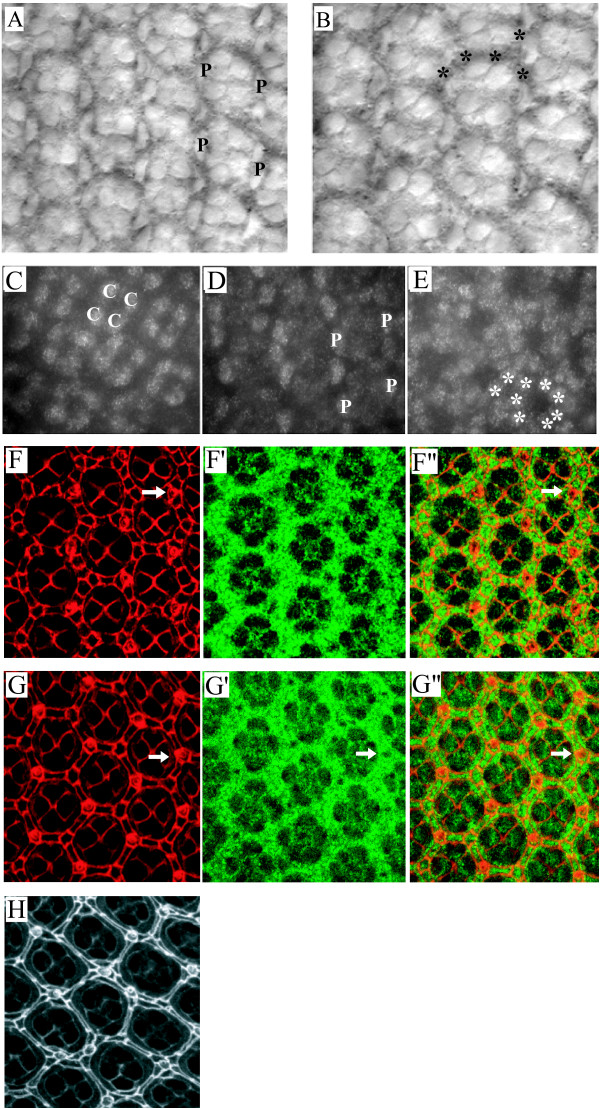
The *echinus *transcript is expressed at low, uniform levels in the pupal eye, and GAL4-driver-dependent expression of ec-SF1 or an *ec*-silencing microRNA suggests that pigment cells are an important site of *ec *action. (A,B) Tissue in situ hybridization of an *echinus *antisense probe complementary to all splice forms in 28 hr APF pupal retinas. (A) Focal plane showing primary pigment cells. (B) Focal plane showing IOCs. (C-E) Pupal retinas from heterozygous *ec*^*PlacZ*^/+ flies showing anti-β galactosidase staining in cone cells (C), primary pigment cells (D), and IOCs (E). Cell types indicated are cones (C), primaries (P), and IOCs (*). (F-F") 24 hr pupal retina from LL54-GAL4; UAS-GFP flies stained with anti-Dlg to outline cell boundaries (F, F") and GFP (F',F") to visualize the LL54-GAL4 expression pattern. (G-G") 29 hr pupal retinas from LL54-GAL4; UAS-GFP flies stained as above. LL54-GAL4 is expressed primarily, if not exclusively in pigment cells, but not bristles or cone cells. (H) 36 hr pupal eye from LL54-GAL4; UAS-CG2904-RNAi stained with anti-Dlg. Extra IOCs and sorting defects are apparent. Arrows indicate bristles.

cDNAs for echinus have been isolated from early embryos [54], as well as pupal eyes (this work), and genetic interactions between *echinus *and genes that result in phenotypes in tissues other than the eye have been described [48,52]. Thus, it is likely that *echinus *is expressed in, and plays roles in tissues other than the eye, though our focus in this work is the pupal eye. To determine the *echinus *expression pattern in this tissue we carried out tissue *in situ *hybridizations on pupal retinas with an antisense *echinus *cDNA probe. *echinus *transcripts could be detected at low levels in cone cells, primary pigment cells and IOCs prior to, and during the period of IOC death (Fig. 3A,B). The *ec*^*PlacZ *^allele carries a version of *lacZ *that functions as an enhancer trap. Therefore, as a second, and perhaps more sensitive method of visualizing *echinus *expression, we examined β-gal expression in pupal retinas from this line. Consistent with the results from *echinus *tissue *in situ *hybridizations, β-gal was expressed uniformly in cone cells, primary pigment cells and IOCs in wildtype (heterozygous *ec*^*PlacZ*^) pupal retinas (Fig. 3C–E). These observations do not exclude the possibility that Echinus protein is differentially translated in specific cells, but they do suggest that transcription of *echinus *in specific populations of IOCs is not a critical point of cell sorting or cell death regulation.

To explore the question of where *echinus *expression was required during pupal eye development we took advantage of a GAL4-driver, LL54-GAL4, that is expressed predominantly, if not exclusively in primary, secondary and tertiary pigment cells, but not cone cells or bristles (Fig. 3F–G) [55]. Expression of LL54-GAL4 in a wildtype background, in conjunction with a UAS-driven miRNA (UAS-CG2904-RNAi) targeting all *echinus *splice forms, phenocopied the *echinus *phenotype (Fig. 3H). We cannot exclude the possibility that low-level expression of the *echinus*-targeting miRNA in other cell types in the eye is sufficient to generate this phenotype. This possibility notwithstanding, our observations suggest that *echinus *normally functions, at least in part, within the pigment cells to regulate IOC fate. Interestingly, however, expression of either of two different splice forms of *echinus *(ec-SF1 and ec-SF2; see below), in an *ec*^*EP*Δ4 ^background, under this same promoter, failed to rescue the *ec*^*EP*Δ4 ^phenotype (data not shown). This observation suggests, but does not prove, that *echinus *expression is also required in other cell types to bring about proper IOC sorting and death. Analysis of *echinus *clones will be required to determine definitively the cell types in which *echinus *expression is required. Finally, GMR-driven transgenes are expressed in all cell types in the developing eye [50,56,57]. As noted above (Fig. 2E,K), forced expression of *echinus *in all retinal cells did not by itself induce defects in sorting or ectopic retinal (IOC) cell death. This, in conjunction with the observation that endogenous *echinus *is expressed uniformly in the pupal retina, is consistent with a model in which *echinus *expression is not sufficient to induce the death of IOCs, though it is necessary.

### Echinus gives rise to multiple splice forms that encode proteins with homology to ubiquitin-specific proteases

We sequenced multiple *echinus *cDNAs and identified three splice variants (designated ec-SF1, ec-SF2, and ec-SF3) (Fig. 1A–C). In each of these, a common 3' coding and UTR sequence is spliced to distinct 5' UTR and coding sequences. To determine which splice forms are expressed during pupal retinal cell death, we conducted RT-PCR using exon-specific primers. We found that all isoforms were expressed in the pupal retina during the stages when IOC death occurred (see Additional File 3). No differences were seen when expression of different splice forms was monitored using tissue in situ hybridizations (data not shown).

Each splice form encodes a large protein of roughly 1700 aa. Blast searches identified only one region of homology with other proteins, an N-terminal USP domain, a domain found in one of the seven families of deubiquitinating enzymes (DUBs). USP-containing DUBs are cysteine proteases that are capable of removing ubiquitin or ubiquitin-like proteins from substrates [58,59]. The USP domain features two short, well-conserved motifs – the Cys box, which contains the essential catalytic cysteine, and a His box, which contains conserved His and Asp residues that are thought to be essential for catalysis. Structural studies on the USPs HAUSP and Ubp14 have revealed that the catalytic histidine and aspartic acid deprotonate the catalytic cysteine allowing for nucleophilic attack [60,61]. Ec-SF1 encodes an USP domain with all the known essential catalytic residues (Figure 1A). Database searches identified genomic sequences that if spliced, would generate similar forms of *echinus *in multiple *Drosophila *species as well as several other insects. Outside of the arthropods Echinus shows most homology with the mammalian DUBs USP53 and USP54, with essentially all of this homology occuring within the USP domain. Most importantly for the purposes of this report, Ec-SF2 and Ec-SF3 encode proteins with truncated USP domains and lack residues important for catalysis. Specifically, Ec-SF2 and Ec-SF3 contain the catalytic histidine and aspartic acid residues found in the USP His box, but they lack the Cys box and its catalytic cysteine. Ec-SF2 and Ec-SF3 instead have alternative N-termini that are conserved in *Drosophilia *and show no sequence similarity to the Cys box motif (Figure 1B, C). The homologous mammalian DUB USP54 also encodes 4 alternative splice forms, two of which do not contain complete USP domains [59].

### Echinus lacks USP activity on a model substrate, and USP activity is not required for echinus-dependent death of IOCs

**Figure 4:**
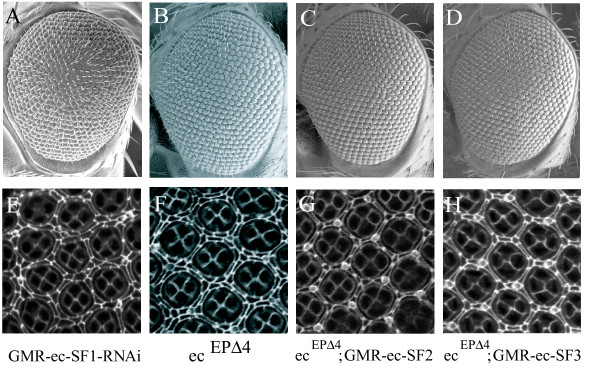
Echinus does not require deubiquitinating activity to promote normal IOC death. (A-D) SEMs of adult eyes of various genotypes. (E-H) Pupal retinas of various genotypes stained with anti-Dlg. (A,E) GMR-driven expression of a microRNA targeting ec-SF1 results in an echinus phenotype. (B,F) *ec*^*EP*Δ4 ^eyes. (C,G) Eyes of genotype *ec*^*EP*Δ4^; GMR-ec-SF2/+. (D,H) Eyes of genotype *ec*^*EP*Δ4^; GMR-ec-SF3/+. Expression of versions of Echinus that lack essential USP catalytic residues rescues the *ec*^*EP*Δ4 ^phenotype.

We generated flies expressing a microRNA that targets Ec-SF1 specifically, under the control of the GMR promoter. These flies showed an *echinus*-like adult rough eye phenotype, and pupal eyes contained extra IOCs (Fig. 4A,E). In contrast, GMR-driven expression of microRNAs designed to target Ec-SF2 or Ec-SF3 resulted in flies that appeared wildtype, and no extra IOCs were observed (data not shown). We cannot exclude the possibility that the microRNAs targeting Ec-SF2 and Ec-SF3 failed to phenocopy *echinus *because they failed to induce a large enough decrease in splice form expression (though in other experiments expression of these miRNAs was sufficient to suppress rescue of *echinus *by GMR-dependent expression of ec-SF2 or ec-SF3; data not shown). Nonetheless, our results from targeting Ec-SF1 demonstrate that this splice form, at least, is important for bringing about IOC death, and suggest that it may be the most important for regulating IOC survival. Also consistent with this hypothesis is our observation that GMR-driven RNAi that targets all splice forms (GMR-CG2904-RNAi) results in a phenotype similar to that observed in flies in which only ec-SF1 was targeted (Fig 2C,I). USP activity per se is unlikely to be sufficient for rescue, since the *echinus *sorting and cell death phenotypes could not be rescued by GMR-dependent expression of yeast UBP2, a ubiquitin-specific protease known to be active on multiple substrates [62,63] (data not shown).

To test the hypothesis that Ec-SF1 has deubiquitinating activity, we measured its ability to cleave a model ubiquitin-linked substrate, Ub-Arg-B-Gal, a fusion protein of Ubiquitin (Ub) and *Eschericia coli *β-galactosidase (β-Gal), separated by an arginine (Arg) residue [62,63]. Ub-Arg-β-gal is a stable protein, and thus bacterial cells expressing it form blue colonies in the presence of the substrate X-Gal (5-bromo-4-chloro-3-indolyl-β-D-galactopyranoside) (Table 2). Deubiquitinating enzymes that remove Ub create Arg-β-gal, an unstable protein. Thus, cells expressing Ub-Arg-β-gal, as well as an active deubiquitinating enzyme such as yeast Ubp2, give rise to white colonies in the presence of X-Gal (Table 2) [62]. Many deubiquitinating enzymes are active in this assay. However, in contrast to yeast Ubp2, expression of ec-SF1 or ec-SF2 in Ub-Arg-β-gal cells, in the presence of X-Gal, resulted in the formation of blue colonies (Table 2). The human proteins most homologous to Ec-SF1, USP53 and USP54, also lack activity in this assay [64]. These results are consistent with models in which Echinus, in particular Ec-SF1, lacks deubiquitinating activity. However, as discussed below (the Discussion), the failure to detect cleavage of a model substrate does not rule out the possibility that Ec-SF1 has activity on other (unknown) substrates.

**Table 2:** Bacterial Assay for the Deubiquitinating Activity of Echinus

Plasmid	Colony Color
pUb-Arg-β-Gal	Blue
pRB105 (yUbp2)	White
pRB-ec (SF1)	White
pRB-ec (SF2)	White
pUb-Arg-β-Gal pRB105 (yUbp2)	White
pUb-Arg-β-Gal pRB-ec (SF1)	Blue
pUb-Arg-β-Gal pRB-ec (SF2)	Blue

Table 2. Echinus ec-SF1 lacks deubiquitinating activity on a model substrate in bacteria. Ubiquitin-Arg-β-Gal has a long half-life, and colonies expressing this protein alone are therefore blue in the presence of X-gal substrate. Expression of *S. cerevisiae *Ubp2 with Arg-β-Gal results in cleavage of ubiquitin, exposing the N-terminus of Arg-β-Gal, which has a short half-life (white colonies). In contrast, expression of ec-SF1 or ec-SF2 does not result in significant cleave Ub-Arg-β-Gal (blue colony color).

To test the hypothesis that Echinus USP activity is required for its ability to bring about the death of excess IOCs, we asked if expression of Echinus splice forms that lack critical USP catalytic residues, Ec-SF2 and Ec-SF3, could rescue the *echinus *phenotype. Somewhat to our surprise, when introduced into the *ec*^*EP*Δ4 ^background (Fig. 4B,F), expression of Ec-SF2 (Fig. 4C,G) or Ec-SF3 (Fig. 4D,H), resulted in complete restoration of normal IOC death. These experiments involve gene overexpression and do not address the question of whether Ec-SF2 or Ec-SF3 is normally required for IOC death. However, they do demonstrate that Echinus USP activity is unlikely to be absolutely required for IOC death, since these splice forms lack residues necessary for this activity.

### Echinus does not show significant genetic interactions with components of the core apoptosis machinery, or other pathways implicated in regulation of retinal cell death

**Figure 5:**
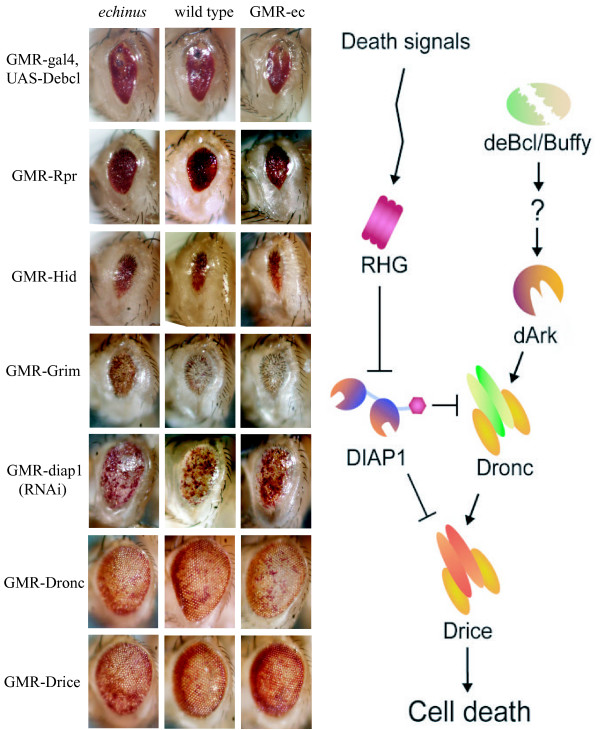
Genetic interactions between *echinus *and known or potential regulators of cell death in the eye. To the right is a schematic depicting known or suggested interactions between death regulators in the fly. The question mark separating Debcl/Buffy from Ark indicates the uncertainy as to the roles these proteins play in regulating Ark activation or activity. GMR-driven transgenes of the indicated genotype were introduced into the *ec*^*EP*Δ4 ^background, or into a wildtype background in the presence of GMR-ec-SF1. For each death regulator tested, similar phenotypes were observed in the presence of GMR-ec-SF2 (data not shown).

Our observations presented in Fig. 2 indicate that null alleles of *echinus *have a previously unappreciated defect in cell sorting as well as cell death. The cell death defects observed in *echinus *mutants could simply be the indirect result of a failure in cell sorting. Alternatively, *echinus *may play roles in both sorting and cell death. We have chosen to explore how *echinus *could be promoting the death of specific IOCs. We searched for genetic interactions between *echinus *loss- and gain-of-function (overexpression) and signaling pathways known to regulate IOC death. GMR-ec phenotypes in Fig. 5 refer to ec-SF1. Similar phenotypes were observed with GMR-ec-SF2 (not shown). The RHG family protein Hid is required for normal IOC death, as are the caspases Dronc and Drice. Loss or overexpression of *echinus *had no significant effect on dominant eye phenotypes associated with GMR-driven overexpression of any of these molecules, or several other cell death activators including Rpr, Grim, Debcl (Fig. 5) or the caspases Dcp-1 and Strica (see Additional File 4). *echinus *loss- and gain-of-function also had no effect on a small eye phenotype associated with a partial loss-of-function in DIAP1 resulting from GMR-dependent expression of dsRNA corresponding to sequences within the *diap1 *coding region (GMR-diap1-RNAi flies) [12] (Fig. 5).

We also failed to see interactions between loss and gain-of-function of *echinus *and mutations in several other pathways implicated in IOC death. These include the following: the EGF pathway (the dominant EGFR allele EGFR^Ellipse^, and GMR-driven versions of Ras, Sina and Yan); the Runx transcription factor lozenge (*lz*^50*e *^and GMR-lozenge); Notch (GMR-GAL4-UAS-Delta, *N*^*fa*-*g*^); and JNK (GMR-GAL4, UAS-dTAK) (see Additional File 4). GMR-GAL4-UAS-klumpfuss was lethal in combination with GMR-ec. However, the significance of this interaction is unclear since expression of GMR-GAL4-UAS-klumpfuss alone resulted in only rare adults (see Additional File 4). Finally, no interactions were observed between loss- or gain-of-function mutations in *echinus *and *rst *(see Additional File 5).

## Discussion

We showed that *echinus*, a gene required for normal IOC death, corresponds to CG2904. CG2904 generates multiple transcripts, each of which encodes a protein with homology to the USP family of ubiquitin-specific proteases. Echinus is necessary but not sufficient to cause IOC death when overexpressed. These results are consistent with models where *echinus *provides an activity that can modulate other signals that drive the death of specific cells (or prevent their survival), rather than providing death signals to specific cells. Previous analyses of the *ec*^1 ^allele led to a model wherein *echinus *functions subsequent to IOC sorting [49,50]. However, our analysis of multiple newly generated *echinus *alleles, including multiple likely null alleles, showed the presence of many excess IOC cells arranged in a side-by-side configuration with respect to primary pigment cells, in addition to excess cells arranged end-to-end. These observations are consistent with a model in which *echinus *functions to promote proper IOC sorting, with the failure in IOC death being a result of incorrectly positioned IOCs being unable to send or receive cell death signals. An alternative possibility that we explored is that *echinus *plays roles in cell death signaling as well as cell sorting. We failed to observe significant genetic interactions between *echinus *and known or suspected death regulators. While these observations do not rule out the possibility that *echinus *acts to regulate death at a novel point, they tend to support models in which *echinus *functions primarily to regulate cell sorting.

Homology searches of genome sequence in other insects suggest that *echinus *is conserved. But nothing is known about the functions of any of these genes. One splice form of *echinus*, ec-SF1, encodes a protein that contains catalytic residues essential for USP activity. ec-SF1 is expressed in the pupal eye and splice form-specific RNAi of this transcript phenocopied *echinus*. However, Ec-SF1 was inactive in a deubiquitination assay utilizing a model ubiquitin-β-gal fusion protein substrate. This may reflect the fact that ec-SF1 is inactive as a USP. Alternatively, Echinus may only be active on specific substrates, a phenomenon observed with a number of USPs [58,59,65]. It is also possible that Ec-SF1, as with several other USP family members, cleaves proteins modified with other ubiquitin-related proteins such as ISG15 [66] or Nedd8 [67]. Regardless of the answer to this question, our observation that splice forms of Echinus that lack residues essential for USP activity rescue the *echinus *phenotype strongly suggests that ubiquitin or ubiquitin-like protease activity is not essential for Echinus function, at least for bringing about the sorting and death of excess IOCs. This does not mean that Echinus functions are necessarily unrelated to regulation of deubiquitination. Interestingly, like *echinus*, the genes USP53 and USP54 in human and mouse have a shorter splice form missing key catalytic residues and a longer form that includes these residues (the longer forms are shown in Fig. 1). Also like Echinus, the USP53 and USP54 long forms are inactive in a deubiquitination assay using a model ubiquitin-β-gal fusion protein substrate [63]. This conserved similarity in gene structure suggests a functional requirement for the multiple splice forms of *echinus*, despite the observed absence of protease activity. Inactive versions of known USPs can, in some cases, still bind ubiquitinated substrates, functioning as dominant negatives that block deubiquitination, thereby facilitating degradation or other events dependent on ubiquitin conjugation [68-71]. A number of components of the ubiquitin pathway regulate cell death in the fly eye, including the ubiquitin activating enzyme uba1, the E3 ubiquitin ligase DIAP1, two components of an SCF-type E3 ubiquitin ligase (*skpA *and a novel F-box gene, *morgue*), and the deubiquitinating enzyme *fat facets *[29,31,32]. Perhaps Echinus promotes cell sorting and death by binding substrates modified with ubiquitin or ubiquitin-like proteins, thereby blocking the removal of these modifications. Alternatively, Echinus could titrate cellular inhibitors of ubiquitin or ubiquitin-like proteases. Finally, it is important to emphasize that Echinus's functions in sorting and cell death may be unrelated to ubiquitin or ubiquitin-like proteins. Some USPs affect signaling through pathways which are independent of their ability to remove ubiquitin or ubiquitin-like proteins [72]. Central to addressing these questions is the identification of proteins bound by Echinus in the eye.

As a first step in this direction we searched for genetic interactions between *echinus *and mutations in genes known or suspected to regulate IOC survival. EGFR or Notch, important upstream regulators of IOC survival. Particularly in the case of the Echinus overexpression experiments, our observations suggest that *echinus *is not sufficient on its own to regulate signaling through these pathways. We also searched for interactions between *echinus *and a number of known or suspected cell death effectors. These included Rpr, Hid, Grim, and the caspases Dronc, Drice, and Dcp-1. For each of these genes, and for the eye-specific partial loss-of-function of DIAP1 induced using GMR-diap1-RNAi, expression in the eye results in ectopic cell death. However, none of these phenotypes were significantly suppressed in the *echinus *loss-of-function background, or enhanced by *echinus *coexpression. Perhaps *echinus *does not regulate these components, or components in the same pathways, at downstream points. However, there are several caveats to this conclusion. First, *echinus *is rare at the level of mRNA, and thus presumably at the level of protein as well. Therefore, it may simply be that loss of *echinus*, which normally regulates its target(s) in the context of their much lower endogenous expression level, has little effect on phenotypes due to high-level target expression. Second, in the cases where Echinus was overexpressed, it may be that *echinus *requires cofactors in order to act on its targets, and these also may be relatively rare and rate-limiting. If true, overexpression of *echinus *might again be expected to have little effect on phenotypes associated with high-level expression of its target proteins. Therefore, our observations allow us to conclude at most that *echinus *is probably not a rate-limiting, or dose-dependent regulator of the above death activators.

## Conclusion

The *echinus *locus encodes multiple splice versions of proteins with homology to USP family proteases. But there is no clear evidence that regulation of ubiquitination is relevant to *echinus's *role in promoting IOC sorting and death. *Echinus *did not show significant genetic interactions with a number of known death regulators, and expression of *echinus *was not sufficient on its own to induce ectopic death. Together these observations suggest several possibilities. The first is that *echinus *regulates – but only in very specific contexts/cell types – unknown (or untested) upstream regulators or effectors of the core cell death pathway. Alternatively, *echinus *may act as a necessary but not sufficient component in a parallel death signaling or effector pathway. Finally, *echinus *may function primarily to regulate cell sorting, with failure in this process leading to cell survival because IOCs are unable to effectively transmit or receive death signals. Drawing links between *echinus *and any of these pathways requires the identification of Echinus targets.

## Methods

### Identification and sequencing of echinus alleles

Adult males were exposed to EMS and mated with females carrying an attached-X chromosome (XX/Y). Progeny males that had rough eyes were crossed back to attached-X females and stocks, designated *ec*^56 ^and *ec*^30^, were established. Complementation tests were used to establish allelism of with *echinus*. Genomic DNA for each EMS allele was isolated from third instar larvae using standard DNA isolation protocols. The coding portions and flanking DNA of *echinus *were amplified by PCR using Platinum Taq polymerase (Invitrogen) and sequenced using the Big Dye terminator cycle sequencing kit (v3.1) on a 3730XL sequencer (ABI). The obtained sequence was compared with the published *Drosophila *genomic sequence. Both strands were completely sequenced for each exon and any ambiguities or mutations were re-sequenced. The P element *ec*^*PlacZ *^was isolated originally as a rough eye mutant mapping to the X chromosome that failed to complement *ec*^1 ^(BA Hay, unpublished). Excision alleles of this element, as well as those of a nearby element, *EP(X)1343*, were generated using standard techniques. Deletion alleles of *echinus *were generated through excision of *EP(X)1343*. Approximately 300 independent excision lines were characterized using primers indicated in Figure 1. Several deletions were identified that removed much of the *echinus *coding region. Two of these, *ec*^*EP*Δ4 ^and *ec*^Δ9^, are indicated in Fig. 1A. The *ec*^3*c*3 ^allele was generated by EMS mutagenesis as previously described [53]. The *ec*^Δ9 ^deletion allele resulted from imprecise excision of a P element insertion within the adjacent gene *roX1 *(H. Kramer, unpublished).

### Isolation of echinus cDNAs

*echinus *cDNAs were isolated from a larval-pupal cDNA library using a probe generated against the UCH domain of CG2904. Several clones were isolated and sequenced. These encoded two different splice forms of *echinus*, ec-SF2 (Genbank AY576488) and ec-SF3 (Genbank DQ418878). To identify *echinus *5' cDNA end sequences we carried out 5' RACE. Total RNA was isolated from *w*^1118 ^pupal eye discs using RNeasy Micro Kit (Qiagen). The 5' RACE System was used according to the manufacturer's protocol (Invitrogen). cDNA was made by reverse transcribing the pupal RNA using an *echinus*-specific primer (5'-GGTCTGCAGCTGCTGGAAAAGTTCC-3'). 5' *echinus *transcripts were isolated first by performing PCR using an anchor primer complementary to the dCTP cap of the cDNA and gene-specific primers (5'-AGATACAGTCTTGGCCACCGCATA-3', 5'-TTGTTGTTGGCGCTACTGCCATAGC-3', and 5'-CCGCATTGATGCACCGATCCCTCTC-3'). Nested PCR followed using the internal *echinus *primers (5'-GAAACGATCGTCGAAAGGCGTCCAA-3', 5'-CCAGTGGGGCATGTGGCAGCGATGT-3', and 5'-CCATTACGGCCAATTCCACGCTGCT-3') and another anchor primer. PCR amplicons were purified using QiaQuick Gel Extraction Kit (Qiagen) and sequenced. This work led to the identification of a third *echinus *splice form, ec-SF1 (Genbank DQ418877), which encodes distinct 5' noncoding and coding sequences.

### RNAi-mediated knockdown of echinus function

We used two approaches to silence *echinus *expression. In the first approach a cDNA fragment was placed into the SympUAST vector, which carries UAS elements on opposite strands flanking the insert [73]. Transgenic flies carrying these constructs were recombined with GMR-Gal4 to generate GMR-Gal4-UAS-ec-RNAi flies. Several segments of the coding region were targeted with this strategy: residues 1237–1505, and 232–398, as defined with respect to the sequence of the ec-SF2 cDNA. We also generated flies expressing GMR-driven artificial microRNAs, using the mir-6.1 backbone, designed to target specific 21 bp sequences within *echinus*. In brief, 22 bp sequences complementary to echinus were substituted into the mir-6.1 precursor stem backbone at the position normally occupied by the mature mir-6 miRNA (C.H. Chen and B.A. Hay, unpublished). The 22 nt sequences targeted either the region surrounding the catalytic cysteine of ec-SF1(residues 562–583; cta agg gac tac tca atg gac c), ec-SF2 (residues 530–551; cta aga agt tct cga gca aaa c), or sequences common to all *ec *transcripts (SF1 residues 4252–4273; gca atg caa aaa tgg atg tag a). These constructs are known as GMR-ec-SF1-RNAi, GMR-SF2-RNAi, and GMR-CG2904-RNAi, respectively.

### Echinus and RH68894 transgenes and expression

The coding regions for two splice versions of *echinus*, ec-SF2 and ec-SF3, that lack UCH domain catalytic residues, were introduced into GMR, generating GMR-ec-SF2 and GMR-ec-SF3, respectively. The coding region for ec-SF1, which contains all known UCH catalytic domain residues, was also introduced into GMR, generating GMR-ec-SF1.

### Drosophila lines and genetics

*Drosophila *strains and crosses were performed at 25°C. Pupal timing is expressed in hours, with the white prepupae stage defined as 0 hours after pupal formation (APF). Pupal dissections were performed at 36 hrs APF unless noted otherwise. The following strains were used: UAS-klumpfuss [74], Ellipse, *ec*^1^, *Notch*^*spl*-1^(Bloomington Stock Center, Indiana University), GMR-ΔN dcp-1 [75], GMR-drice [76], *ec*^*PlacZ *^(this work), GMR-hid, GMR-rpr [77], GMR-grim, GMR-dronc [6], GMR-strica and GMR-Gal4-UAS-debcl [78] and GMR-Gal4-UAS-dTak1 [79]. The *echinus *deletion allele mutant *ec*^*EP*Δ4 ^was generated by imprecise excision of the P element insertion line *EP(X)1343*. LL54-GAL4-expressing flies were obtained from Craig Montell [55]. GMR-yUbp2 (GenBank M94916) was cloned into NotI-StuI of pGMR-1N. RH68894 (Research Genetics/Invitrogen) was cloned into the GMR vector.

### Echinus expression pattern

A ~1.2 kilobase region within *echinus *(residues 1,532 to 2,691 with respect to ec-SF2) was amplified by PCR using T3/T7-tailed primers. Digoxigenin-labeled RNA probes were prepared for both sense and antisense strands (Roche). *In situ *hybridization to OreR pupal retinas was performed essentially as described [80]. Pupal retinas from *ec*^Δ9 ^were used as a negative control for staining.

### RT-PCR analysis of echinus splice form expression

Retinas from OreR pupae (26–27 hrs APF) were dissected into PBS and then transferred immediately to RNAlater (Ambion). Total RNA was extracted using Trizol (Invitrogen). RT-PCR Reactions were performed (n = 3) using the SYBR Green One-step RT-PCR reagent kit on an Applied Biosystems 7900 Sequence Detection System. Each 15 μl reaction included 50ng of total RNA and 0.1 μM of each primer. The *echinus *and *rp49 *primer pairs were designed using Primer Express Version 2.0 software (Applied Biosystems) and were constructed to span an intron. Gel electrophoresis melting curve analysis was performed for each run to ensure there was a single major product corresponding to the predicted size and melting temperature. Primer sequences: SF1 splice form: 5'-TGCTTTCTCAATTGTGCCGT-3', SF2 splice form: 5'-CAACATTGGCGCATTCTTTC-3', common 3' primer for SF1 and SF2: 5'-AAGATACAGTCTTGGCCACCG-3', SF3 splice form: 5'-GCCTTGTGCCTGCAAAAGTT-3', 5'-TCAGAGTCACAACATGGCAGC-3', all echinus splice forms: 5'-CAGCTGCCCTTCACCCA-3', 5'-TATGTCGCCCATGTTGCC-3', rp49: 5'-AGTCGGATCGATATGCTAAGCT-3', 5'-AGATACTGTCCCTTGAAGCGG-3'.

### Microscopy, immunocytochemistry, and antibodies

Scanning electron microscope images were produced on a Hitachi machine. Flies were dehydrated in an ethanol series, incubated in hexamethyldisilazane (Sigma) overnight, and dried prior to use. Pupal retinas were dissected in PBS and fixed for 30 minutes in 4% paraformaldehyde. Immunostaining was carried out in PBT (PBS + 0.1% Triton-X100) containing 10% fetal calf serum. Antibodies were used at the following concentration: mouse anti-Dlg (1:150) and mouse anti-β galactosidase (1:15) (Developmental Studies Hybridoma Bank, University of Iowa, Iowa City, IA), rabbit cleaved caspase-3 (1:100, Cell Signaling Technologies). Secondary antibodies included mouse Alexa Fluor 488 (Molecular Probes) and mouse or rabbit IgG conjugated to Cy3 (Jackson Labs). Pupal retinas were mounted in either VectaShield medium (Vector, Burlingome, CA) or Antifade (Molecular Probes).

### TUNEL staining

Pupal retinas were dissected in PBS and fixed for 30 minutes in 3% paraformaldehyde. The *In situ *Cell Death Detection Kit (Roche Applied Science) or Promega DeadEnd Fluorometric TUNEL system was used for TUNEL labeling with fluorescein-dUTP. Tissues were incubated at 37°C for 1 hour in the mixture of enzyme and label solution then rinsed in PBS. Labeled tissues were mounted in Antifade reagent (Molecular Probes), viewed with a Zeiss Axioplan 2 and images were captured using a Retiga 1350EX digital camera (Qimaging Corp.) and Northern Eclipse software (Empix Imagin, Inc.).

### Interommatidial cell counts

Interommatidial cell counts were made by counting the IOCs, minus the bristles, that surround two primary pigment cells. Three separate areas were counted per pupal retina, and at least five pupal retinas were counted for each genotype.

### Deubiquitination assay

Deubiquitination assays were carried out as described in [62].

## Authors' contributions

JMC carried out most of the experiments in this study. IB and JDF isolated and sequenced *echinus *alleles, and carried out tissue-in-situ hybridization and RT-PCR analysis of *echinus *expression. MG participated in data analysis, experimental design and writing of manuscript. SG and BAH were responsible for overall experiment design, analysis of data and, in conjunction with JMC, writing of the manuscript. All authors read and approved the final manuscript.

## Supplementary Material

Additional file 1 *echinus *mutants have a decrease in IOC apoptosis. TUNEL staining of (A) *OreR*, (B) *ec*^*PlacZ *^and (C) *ec*^Δ9 ^pupal retinas (29–30 hr APF). Anti-active caspase-3 immunostaining in (D) *OreR *and (E) *ec*^56 ^and (F) *ec*^*EP*Δ4 ^pupal retinas (30 hr APF). Apoptosis is reduced, though not completely absent, in *ec *mutant pupal retinas. Click here for file

Additional file 2 Phenotype of the *ec*^3*c*3 ^allele in trans to a deficiency for the region containing *echinus*. Scanning electron micrographs and pupal retinas of several different genotypes are shown. *ec*^3*c*3 ^placed in trans to a deficiency that removes *echinus*, *Df(1)HC244*, shows a more severe rough eye phenotype than homozygous *ec*^3*c*3 ^flies. Pupal retinas show a significant increase in the number and improper sorting of IOCs. This genetic observation suggests *ec*^3*c*3 ^represents a partial loss-of-function allele. Click here for file

Additional file 3 Three *echinus *splice forms, ec-SF1, ec-SF2 and ec-SF3, are expressed in the pupal retina during the stage of IOC death. Gel image shows the results of RT-PCR analysis using primers for a positive control (rp49), primers specific to each *echinus *splice form (SF1, SF2, SF3), and primers that recognize all three splice forms (all). M= Size Marker; – and + designate the absence or presence of pupal retinal RNA template; expected product sizes are as follows: 91 bp (rp49), 104 bp (SF1), 111 bp (SF2), 147 bp (SF3), 135 bp (all). Click here for file

Additional file 4 Genetic interactions between *echinus *and components of several signaling pathways. The indicated genotypes were introduced into the *ec*^Δ4 ^background, or into a wildtype background in the presence of GMR-ec-SF1. For each genotype tested, similar phenotypes were observed in the presence of GMR-ec-SF2 (data not shown). Genotypes not discussed in the text are indicated below. The *EGFR*^*ELP *^mutation is a hypermorphic allele of the EGF receptor [81,82]. However, genetically it behaves as a partial loss-of-function allele in the eye because it induces the expression of high levels of the EGFR inhibitor Argos [83]. Downstream Ras pathway components tested include Ras^N17 ^(a dominant negative version of Ras driven by the sevenless promoter) Sina and Yan^act ^(a version of Yan that is not inhibited by MAPKinase phosphorylation). GMR-GAL4-UAS-Delta expresses the Notch ligand Delta in every cell behind the morphogenetic furrow [84]. *N*^*fa*-*g *^removes Notch activity specifically in pigment cells [85], leading to a failure of 1° pigment cells to differentiate, and a decrease in IOC death [86]. *Notch *and *echinus *are both on the X chromosome. To search for interactions between *N*^*fa*-*g *^and *echinus *loss-of-function mutations we took advantage of flies carrying an autosomal insertion of GMR-CG2904-RNAi that targets transcript sequences common to all *echinus *splice forms, and that phenocopies *echinus *(Fig. 2C,I). GMR-ΔN-DCP1 expresses under GMR control a version of the caspse DCP-1 that lacks the N-terminal prodomain. GMR-GAL4-UAS-dTAK flies express under GMR control the kinase TAK1, an activator of JNK signaling. GMR-Strica flies express the long prodomain caspase under GMR control. Click here for file

Additional file 5 *echinus *does not interact with *roughest*. Eye-specific Gain-(GMR-ecSF1) and loss-of-function (GMR-ec-RNAi) of *echinus *mutants were introduced into gain (GMR-GAL4-UAS-*rst*) and loss-of-function (*rst*^*CT*^) *roughest *mutant backgrounds. No significant interactions were observed between these genes. Click here for file
