# Supplementary figures and images for: echinus, required for interommatidial cell sorting and cell death in the Drosophila pupal retina, encodes a protein with homology to ubiquitin-specific proteases
Source: BMC Dev Biol. 2007 Jul 5;7:82. doi: 10.1186/1471-213X-7-82 (PMC1950886; doi:10.1186/1471-213X-7-82)

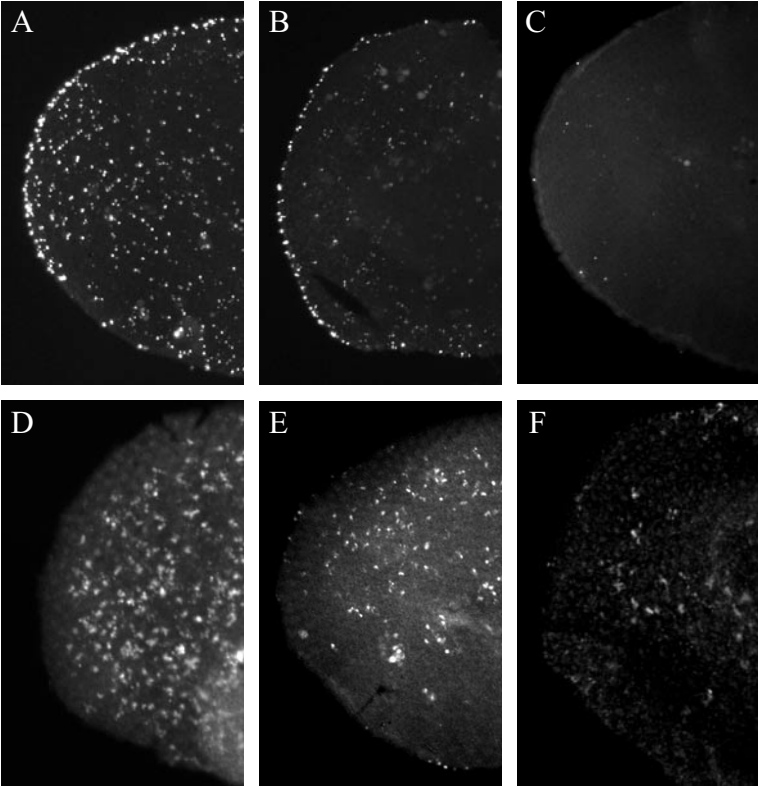

Supplement: Additional file 1 — echinus mutants have a decrease in IOC apoptosis. TUNEL staining of (A) OreR, (B) ecPlacZ and (C) ecΔ9 pupal retinas (29–30 hr APF). Anti-active caspase-3 immunostaining in (D) OreR and (E) ec56 and (F) ecEPΔ4 pupal retinas (30 hr APF). Apoptosis is reduced, though not completely absent, in ec mutant pupal retinas. [file 1471-213X-7-82-S1.pdf]

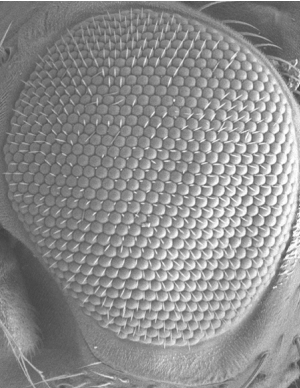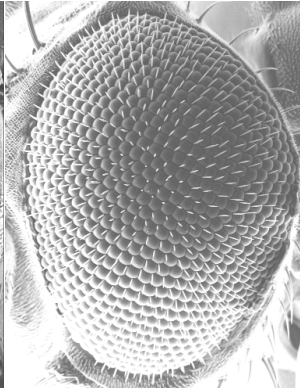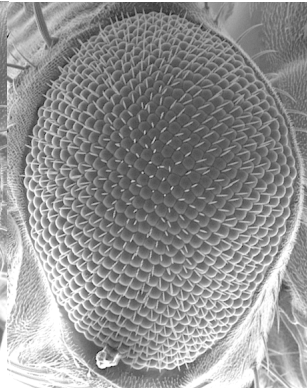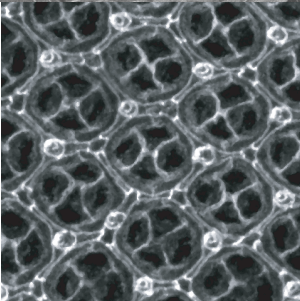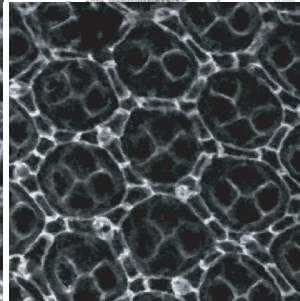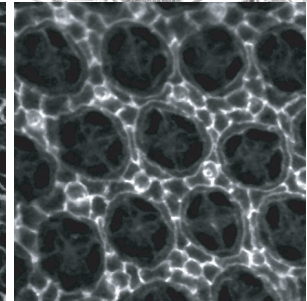

w-

*ec<sup>3c3</sup>*

*ec<sup>3c3</sup>/Df(1)HC244*

Supplement: Additional file 2 — Phenotype of the ec3c3 allele in trans to a deficiency for the region containing echinus. Scanning electron micrographs and pupal retinas of several different genotypes are shown. ec3c3 placed in trans to a deficiency that removes echinus, Df(1)HC244, shows a more severe rough eye phenotype than homozygous ec3c3 flies. Pupal retinas show a significant increase in the number and improper sorting of IOCs. This genetic observation suggests ec3c3 represents a partial loss-of-function allele. [file 1471-213X-7-82-S2.pdf]

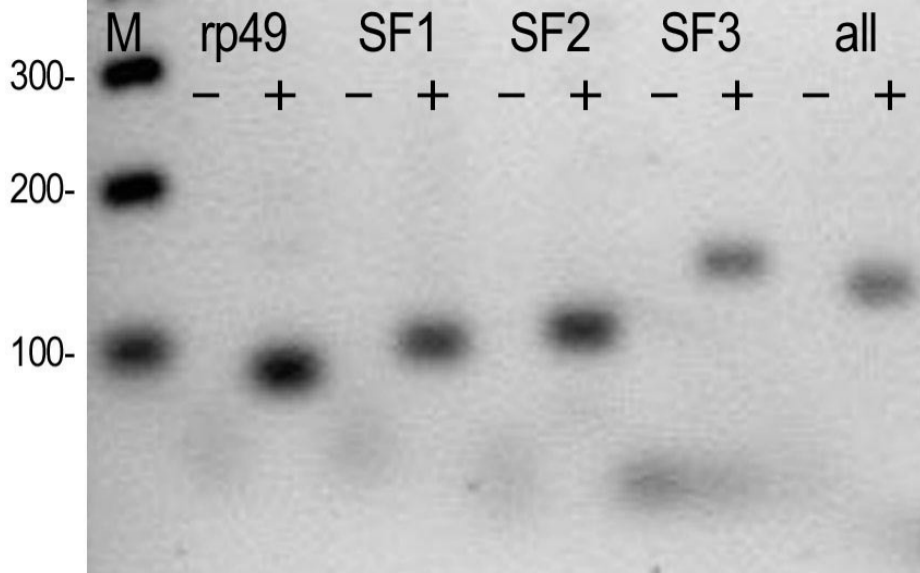

Supplement: Additional file 3 — Three echinus splice forms, ec-SF1, ec-SF2 and ec-SF3, are expressed in the pupal retina during the stage of IOC death. Gel image shows the results of RT-PCR analysis using primers for a positive control (rp49), primers specific to each echinus splice form (SF1, SF2, SF3), and primers that recognize all three splice forms (all). M= Size Marker; – and + designate the absence or presence of pupal retinal RNA template; expected product sizes are as follows: 91 bp (rp49), 104 bp (SF1), 111 bp (SF2), 147 bp (SF3), 135 bp (all). [file 1471-213X-7-82-S3.pdf]

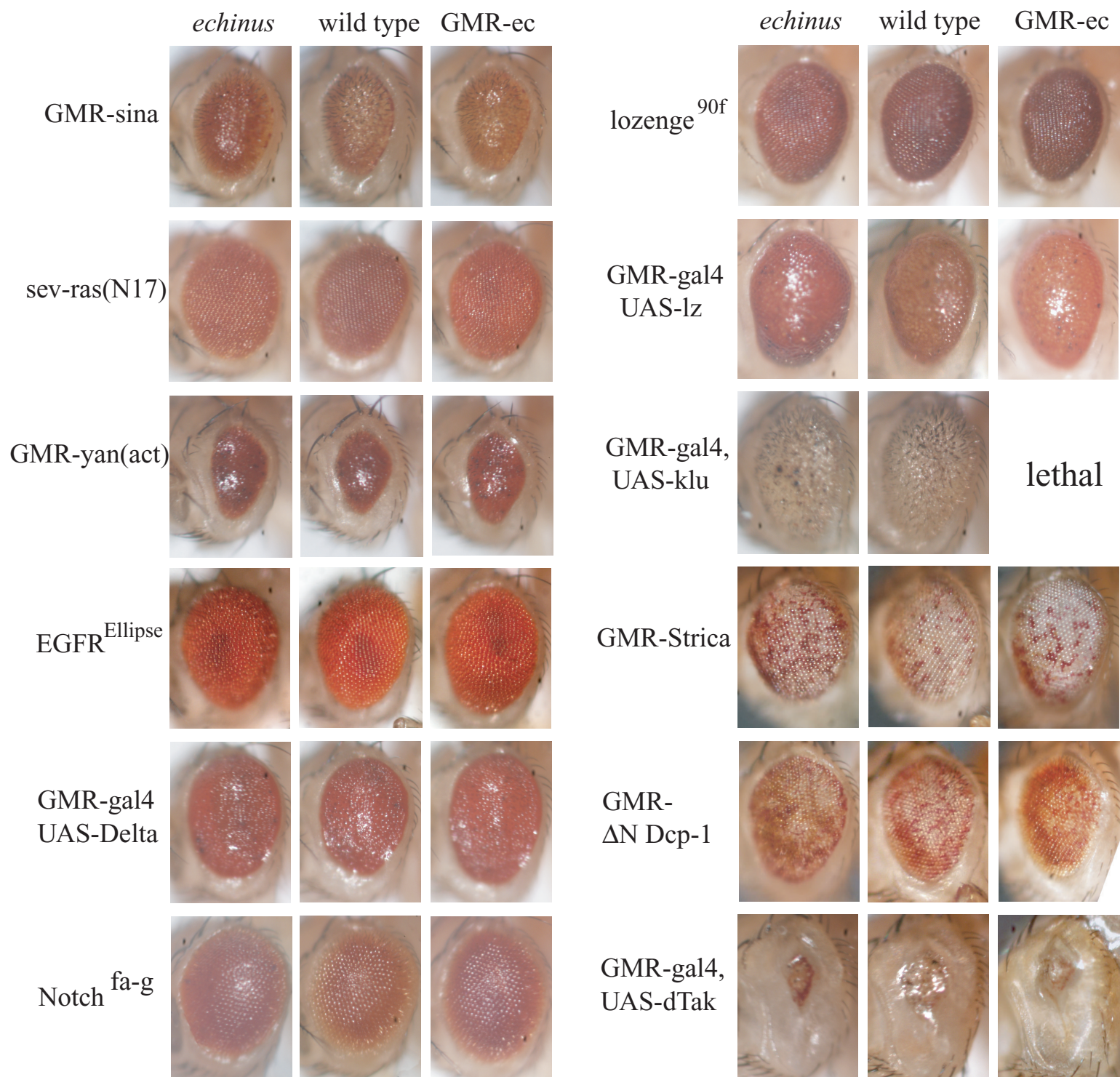

Supplement: Additional file 4 — Genetic interactions between echinus and components of several signaling pathways. The indicated genotypes were introduced into the ecΔ4 background, or into a wildtype background in the presence of GMR-ec-SF1. For each genotype tested, similar phenotypes were observed in the presence of GMR-ec-SF2 (data not shown). Genotypes not discussed in the text are indicated below. The EGFRELP mutation is a hypermorphic allele of the EGF receptor [81,82]. However, genetically it behaves as a partial loss-of-function allele in the eye because it induces the expression of high levels of the EGFR inhibitor Argos [83]. Downstream Ras pathway components tested include RasN17 (a dominant negative version of Ras driven by the sevenless promoter) Sina and Yanact (a version of Yan that is not inhibited by MAPKinase phosphorylation). GMR-GAL4-UAS-Delta expresses the Notch ligand Delta in every cell behind the morphogenetic furrow [84]. Nfa-g removes Notch activity specifically in pigment cells [85], leading to a failure of 1° pigment cells to differentiate, and a decrease in IOC death [86]. Notch and echinus are both on the X chromosome. To search for interactions between Nfa-g and echinus loss-of-function mutations we took advantage of flies carrying an autosomal insertion of GMR-CG2904-RNAi that targets transcript sequences common to all echinus splice forms, and that phenocopies echinus (Fig. 2C,I). GMR-ΔN-DCP1 expresses under GMR control a version of the caspse DCP-1 that lacks the N-terminal prodomain. GMR-GAL4-UAS-dTAK flies express under GMR control the kinase TAK1, an activator of JNK signaling. GMR-Strica flies express the long prodomain caspase under GMR control. [file 1471-213X-7-82-S4.pdf]
