# Supplementary material for: echinus, required for interommatidial cell sorting and cell death in the Drosophila pupal retina, encodes a protein with homology to ubiquitin-specific proteases
Source: BMC Dev Biol. 2007 Jul 5;7:82. doi: 10.1186/1471-213X-7-82 (PMC1950886; doi:10.1186/1471-213X-7-82)
Supplement: Additional file 5 — echinus does not interact with roughest. Eye-specific Gain-(GMR-ecSF1) and loss-of-function (GMR-ec-RNAi) of echinus mutants were introduced into gain (GMR-GAL4-UAS-rst) and loss-of-function (rstCT) roughest mutant backgrounds. No significant interactions were observed between these genes. [file 1471-213X-7-82-S5.pdf]

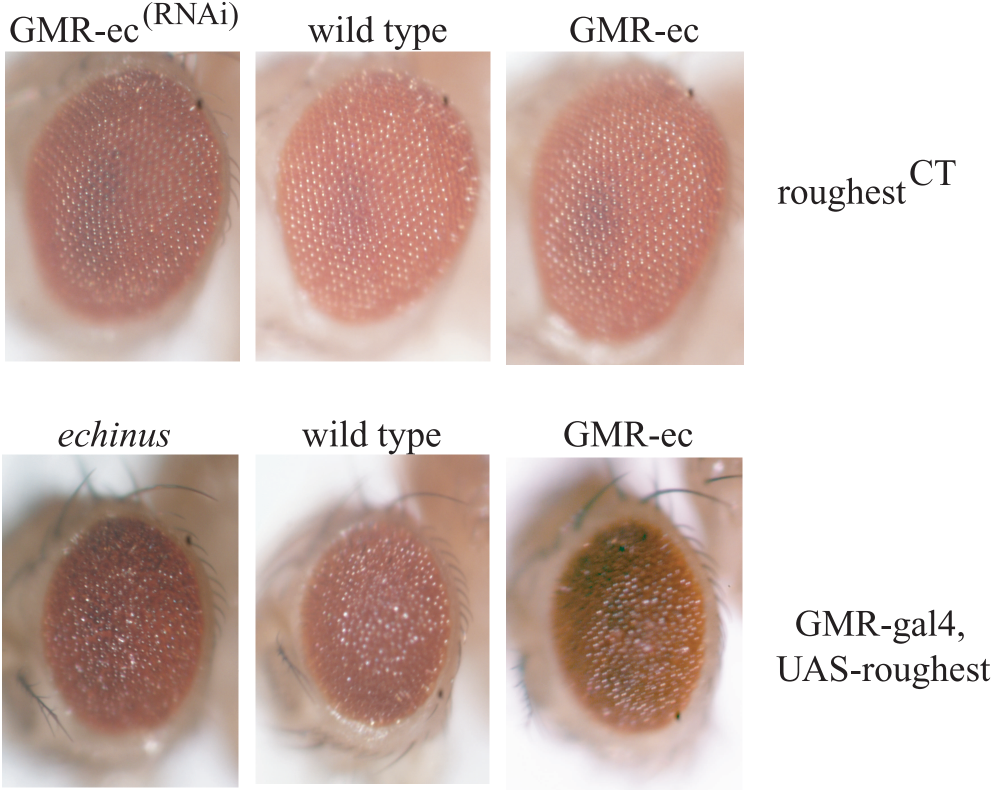
GMR-ec(RNAi)
wild type
GMR-ec
roughest CT
echinus
wild type
GMR-ec
GMR-gal4, UAS-roughest
